# Supplementary material for: The structure of EXTL3 helps to explain the different roles of bi-domain exostosins in heparan sulfate synthesis
Source: Nat Commun. 2022 Jun 8;13:3314. doi: 10.1038/s41467-022-31048-2 (PMC9178029; doi:10.1038/s41467-022-31048-2)
Supplement: Supplementary file 1 — Supplementary Information [file 41467_2022_31048_MOESM1_ESM.pdf]

## Supplementary Information

# The structure of EXTL3 helps to explain the different roles of bi-domain exostosins in heparan sulfate synthesis

L. F. L. Wilson, T. Dendooven, S. W. Hardwick, A. Echevarría-Poza, T. Tryfona, K. B. R. M. Krogh, D. Y. Chirgadze, B. F. Luisi, D. T. Logan, K. Mani, P. Dupree

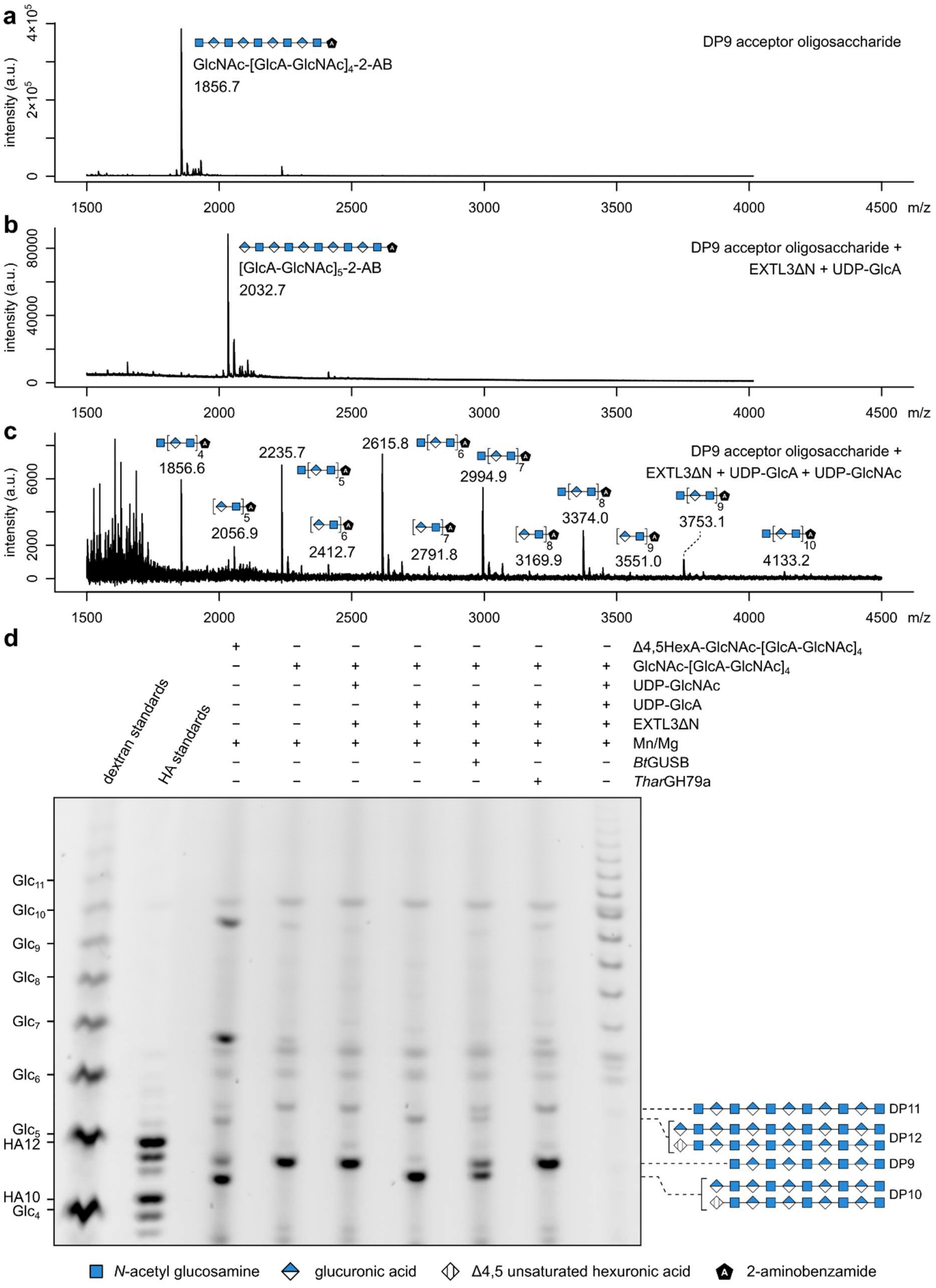


**Supplementary Fig. 1**

**Detailed characterisation of GlcAT-II products by MALDI-TOF mass spectrometry and PACE.** DP9 GlcNAc-[GlcA-GlcNAc]$_4$ acceptor was incubated with a combination of

EXTL3ΔN, UDP-GlcA, UDP-GlcNAc, $MnCl_2$, and/or $MgCl_2$ in an overnight reaction. **a–c** MALDI-TOF mass spectrometry of the reaction products. After reaction termination, products were derivatised at the reducing end with 2-aminobenzamide before analysing by MALDI-TOF. Peaks correspond to deprotonated adducts. Individual spectra correspond to: **a** untreated DP9 GlcNAc-$[GlcA\text{-}GlcNAc]_4$ acceptor; **b** DP9 acceptor incubated with EXTL3ΔN, UDP-GlcA, $MnCl_2$, and $MgCl_2$; **c** DP9 acceptor incubated with EXTL3ΔN, UDP-GlcA, UDP-GlcNAc, $MnCl_2$, and $MgCl_2$. **d** PACE analysis of reaction products (reaction components as indicated). Products visible in lanes 7 and 8 were treated with β-glucuronidase (bovine *Bt*GUSB or *Thar*GH79a from *Trichoderma harzianum*) after reaction termination. Following enzymatic reactions, all products were derivatised at the reducing end with a fluorophore and separated by carbohydrate electrophoresis. HA = hyaluronic acid; HA10 = GlcA-β1,3-[-GlcNAc-β1,4-GlcA-β1,3-$]_4$-GlcNAc, HA12 = GlcA-β1,3-[-GlcNAc-β1,4-GlcA-β1,3-$]_5$-GlcNAc. Results representative of two independent experiments.

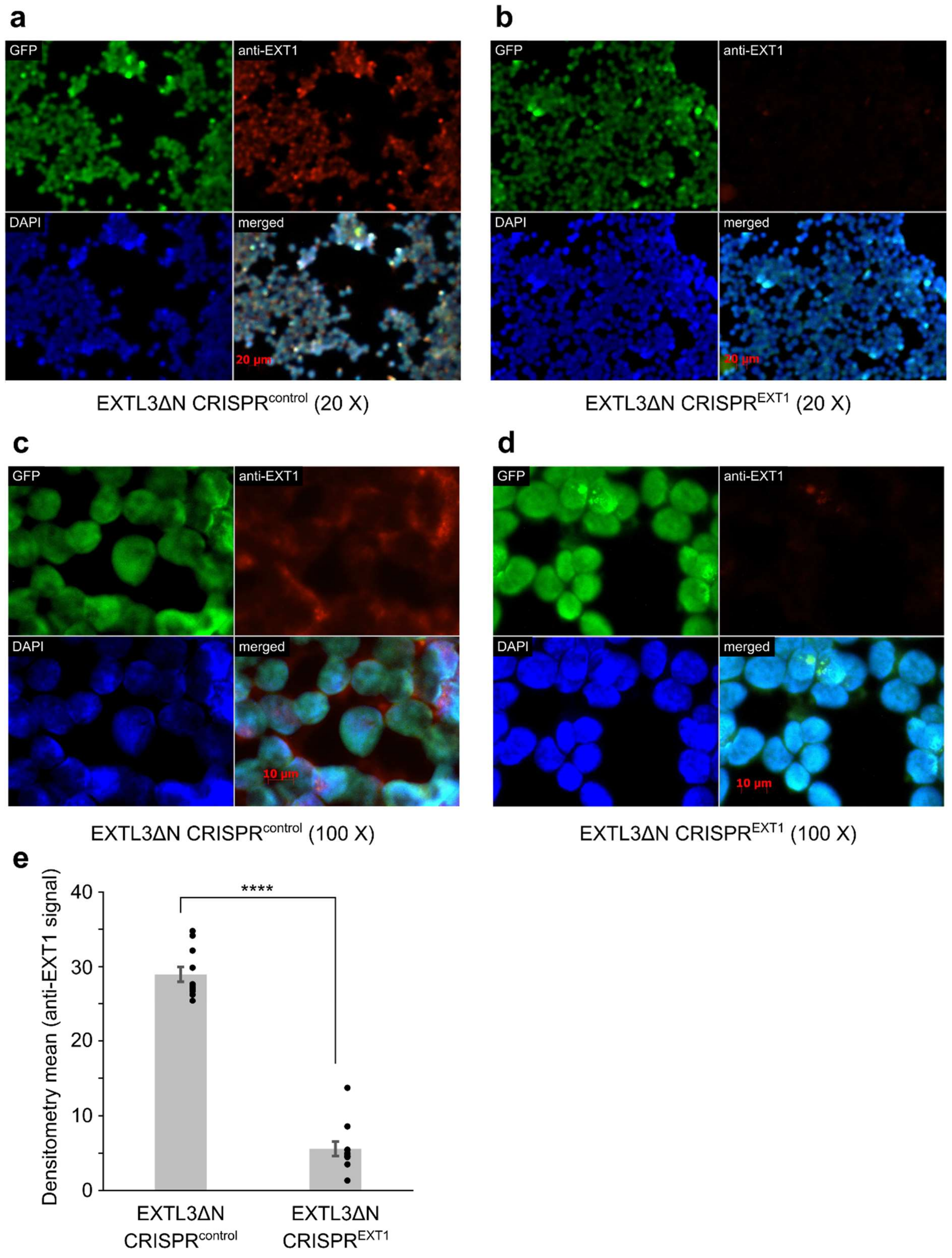


**Supplementary Fig. 2**

**Depletion of endogenous EXT1 by CRISPR-Cas9, as measured by immunofluorescence microscopy.** EXTL3ΔN-expressing EBNA 293 cells were transfected with either a CRISPR/Cas9 non-specific construct (not targeting any known gene; EXTL3ΔN

$CRISPR^{control}$), or a trio of CRISPR-Cas9 constructs targeting EXT1 (EXTL3ΔN $CRISPR^{EXT1}$). All constructs encoded a GFP marker to indicate successful transfection. Cells were subsequently fixed in acetone and stained for EXT1 using anti-EXT1 primary antibody and Alexa Fluor 594-tagged goat anti-mouse IgG. The expression of CRISPR-Cas9 constructs (GFP) and silencing of EXT1 (Alexa Fluor 594) was detected by fluorescence microscopy. Representative cells are shown for EXTL3ΔN $CRISPR^{control}$ (**a**, 20 X magnification; **c**, 100 X magnification) and EXTL3ΔN $CRISPR^{EXT1}$ (**b**, 20 X magnification; **d**, 100 X magnification). Results representative of two biologically independent experiments (total of 11 image stacks). **e** EXT1 immunofluorescence signal in the images was quantified by densitometry and compared between the two treatments. The amount of immuno-reactive EXT1 was significantly lower in the EXTL3ΔN $CRISPR^{EXT1}$ cells compared to the EXTL3ΔN $CRISPR^{control}$ cells (Student's *t*-test, two-tailed, $N = 22$ technical measurements collected over the course of two biologically independent experiments, $p = 2.49 \times 10^{-13}$). Error bars show standard error of the mean. * $p < 0.05$, ** $p < 0.01$, *** $p < 0.001$, **** $p < 0.0001$.

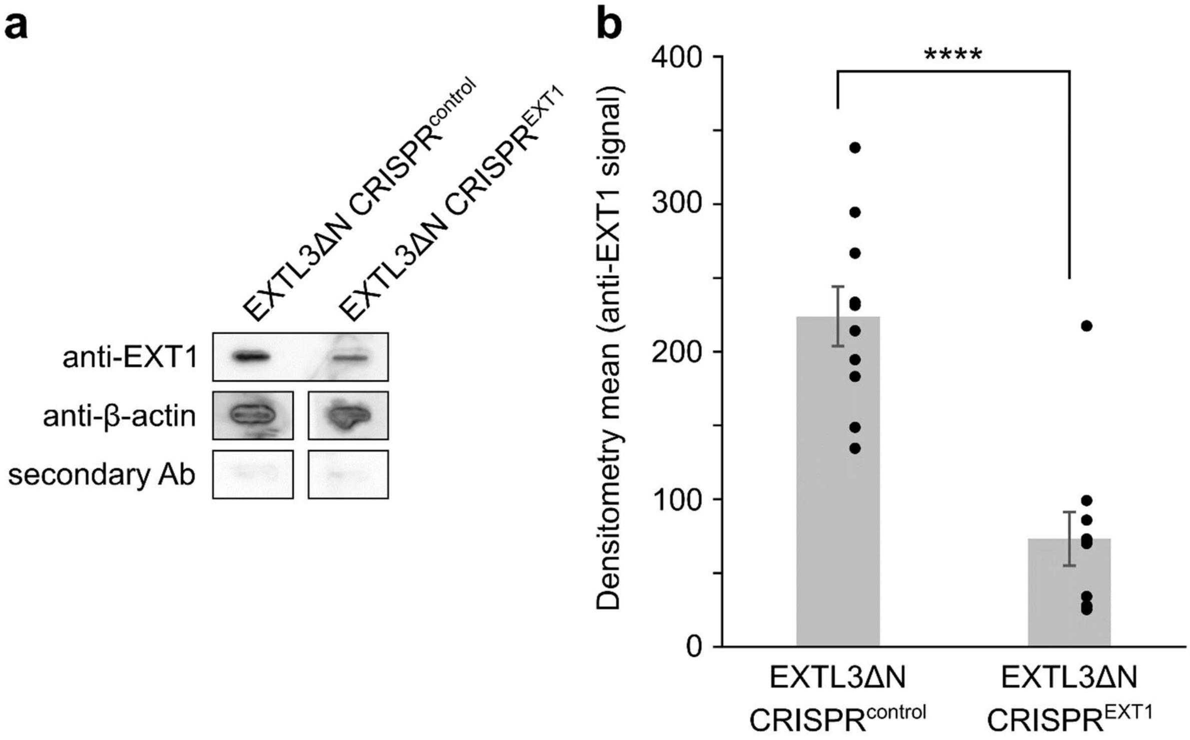


**Supplementary Fig. 3**

**Depletion of endogenous EXT1 by CRISPR-Cas9, as measured by slot blot.** EXTL3ΔN-expressing EBNA 293 cells were transfected with either a CRISPR-Cas9 non-specific construct (EXTL3ΔN $CRISPR^{control}$), or a CRISPR-Cas9 construct targeting EXT1 (EXTL3ΔN $CRISPR^{EXT1}$). **a** Cell extracts were blotted onto PVDF membranes and probed using an EXT1 primary antibody followed by a horseradish peroxidase-conjugated anti-mouse IgG. Loading consistency was verified by probing for β-actin using an anti-β-actin primary antibody. Primary antibody was omitted in the negative control ('secondary Ab'). Results representative of two biologically independent experiments (total of 10 slots for both treatments). **b** EXT1 signal in the blots was quantified by densitometry and compared between the two samples. The amount of immuno-reactive EXT1 was significantly lower in the EXTL3ΔN $CRISPR^{EXT1}$ cells compared to the EXTL3ΔN $CRISPR^{control}$ cells (Student's *t*-test, two-tailed, $N = 20$ technical measurements collected over the course of two biologically independent experiments, $p = 2.86 \times 10^{-5}$). Error bars show standard error of the mean. * $p < 0.05$, ** $p < 0.01$, *** $p < 0.001$, **** $p < 0.0001$.

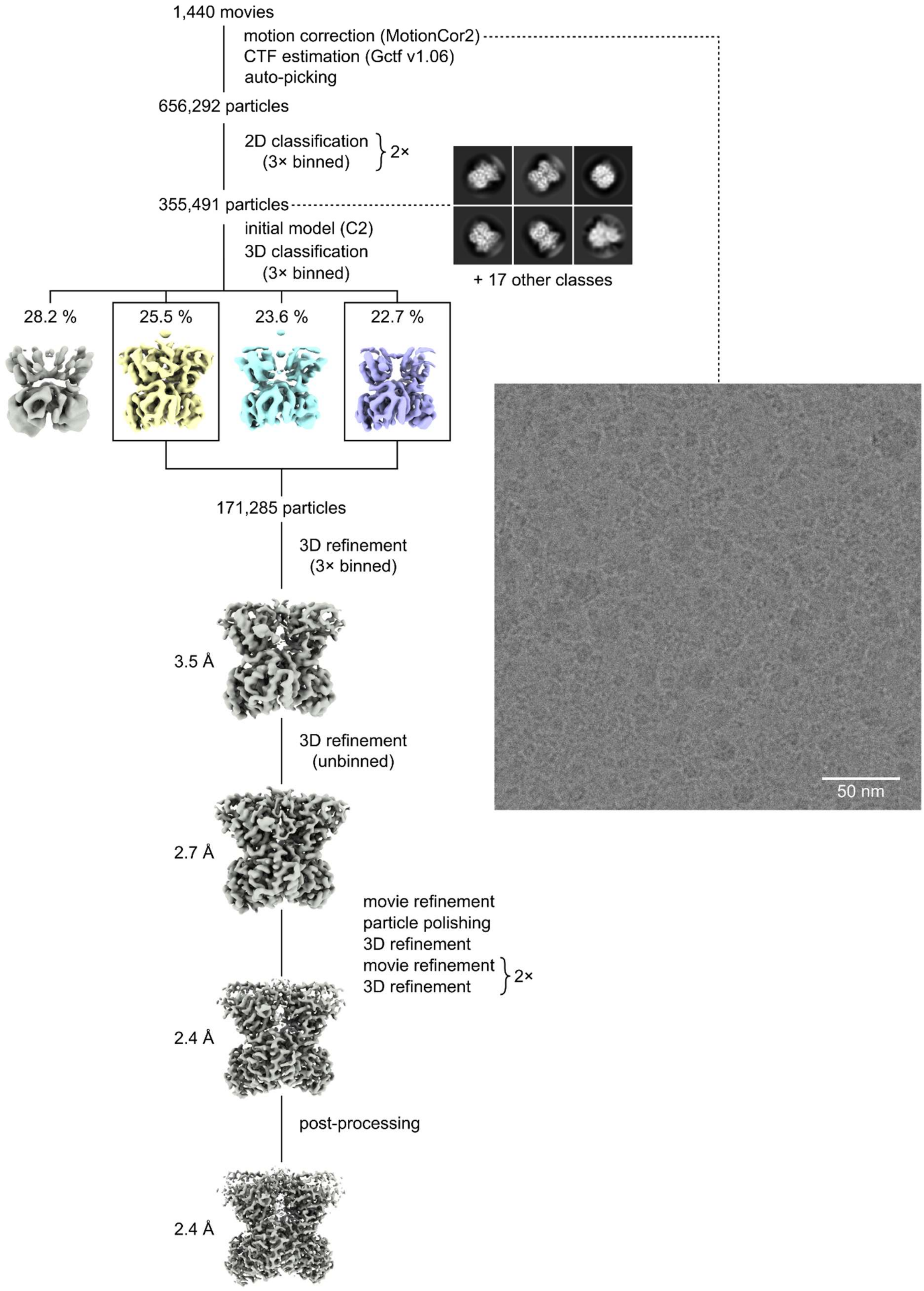


**Supplementary Fig. 4**

**Data processing pipeline for apo-EXTL3 structure.** A representative motion-corrected micrograph is displayed on the right-hand side.

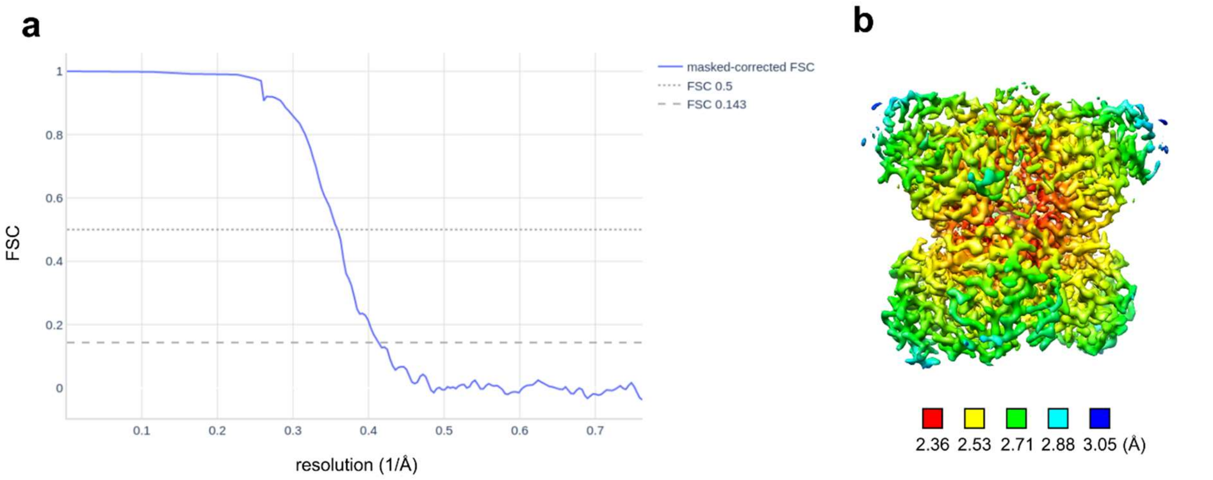


**Supplementary Fig. 5**

**Resolution estimates of the apo-map. a** FSC curve. **b** Local resolution estimate, calculated using RELION, coloured from red (highest resolution) to blue (lowest resolution).

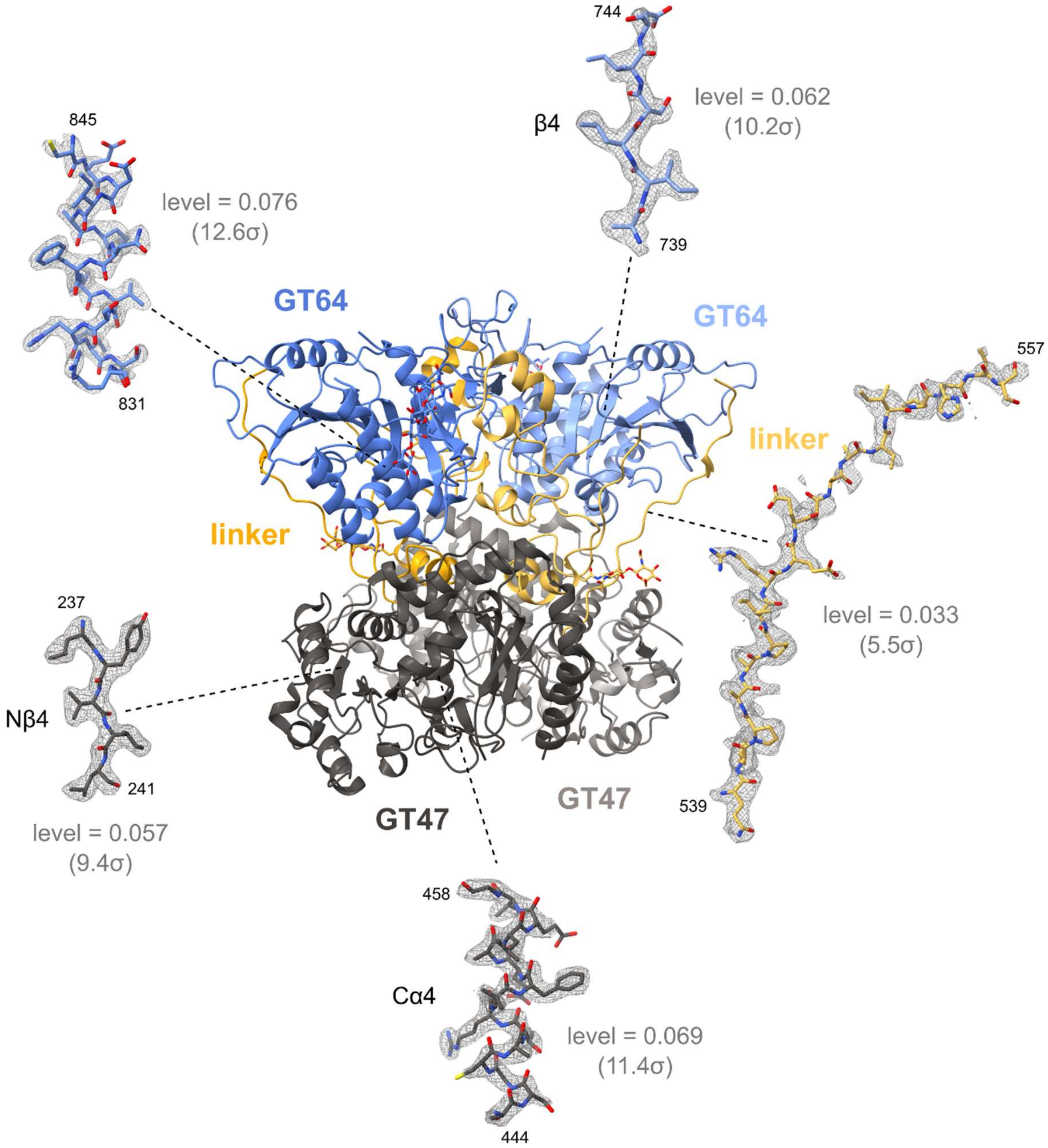


**Supplementary Fig. 6**

**Structure of apo-EXTL3, showing map density in key regions.** GT47 domains: grey; linker regions: yellow; GT64 domains: blue. Each portion of the map is displayed at the indicated contour level.

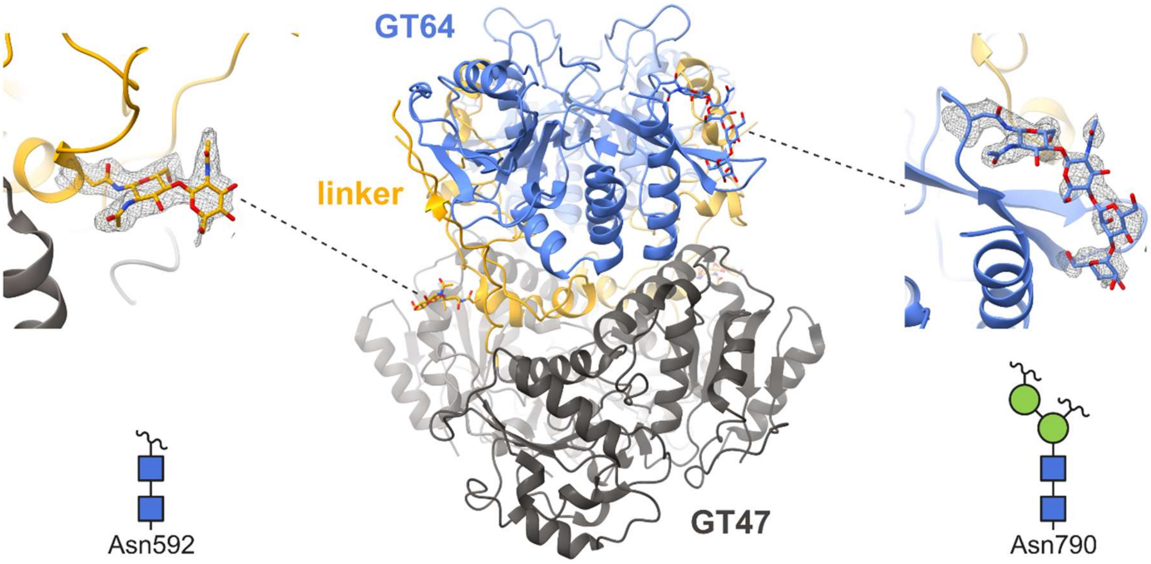


**Supplementary Fig. 7**

**Verification of potential *N*-glycosylation sites in EXTL3.** Asn277, Asn290, Asn592, and Asn790 all possess a Ser/Thr residue at the 2+ position. We previously showed that Asn290 and Asn592 can be decorated with *N*-glycans (variably and invariably, respectively)[26]. EM map density is shown for Asn592 and Asn790, at which N-glycan density was clearly discernible. GT47 domains: grey; linker regions: yellow; GT64 domains: blue. The map densities are shown at a contour level of 0.030 (Asn592; 5.0σ) or 0.027 (Asn790; 4.5σ).

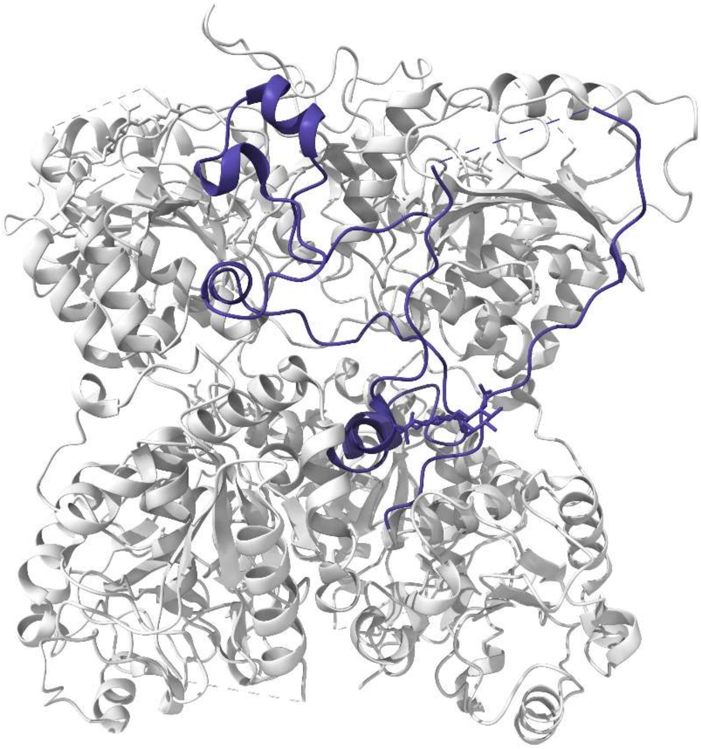

**Supplementary Fig. 8**

**The linker region that connects the GT47 and GT64 domains forms a 'cradle' around the GT64 domain.** The linker region is highlighted in purple.

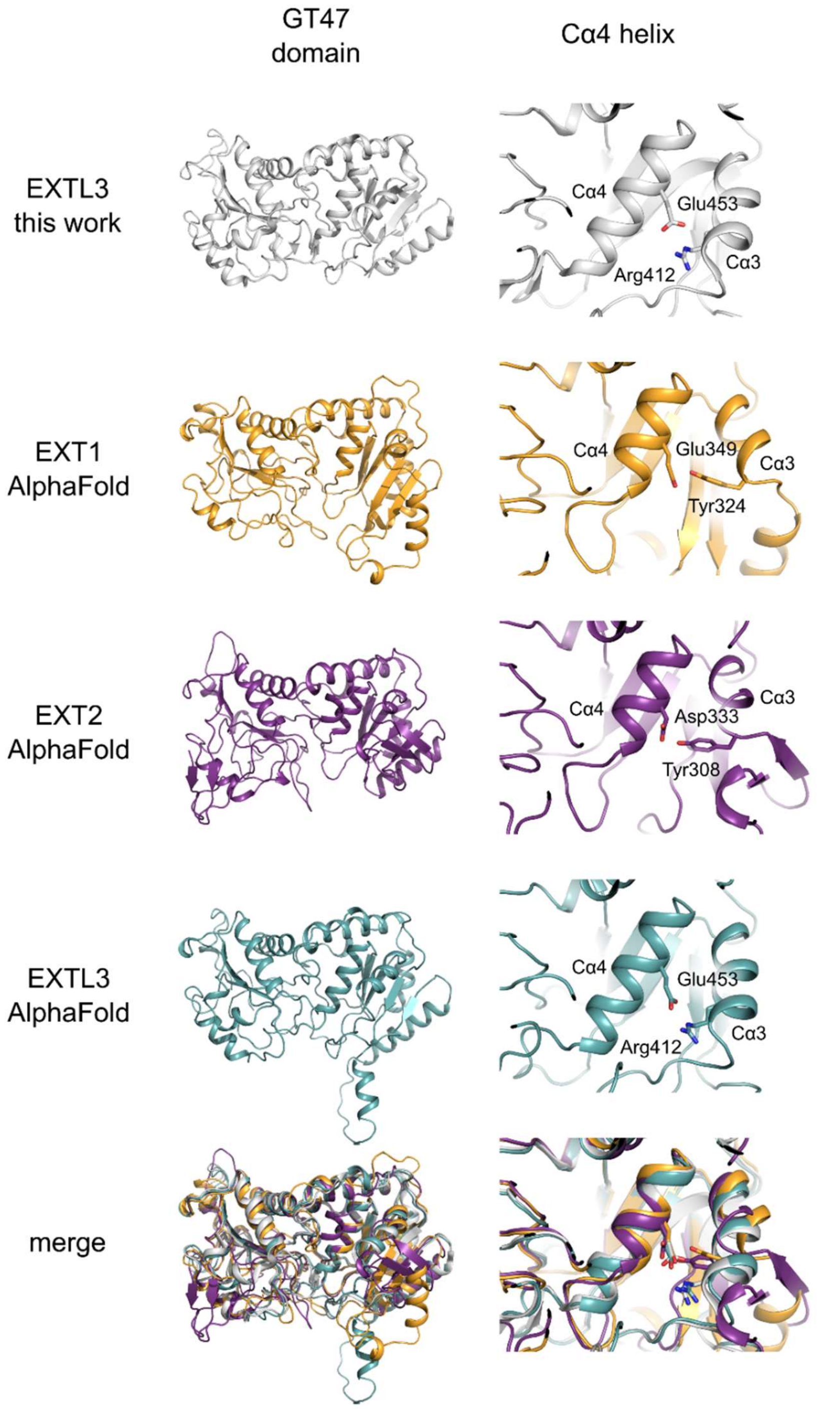


**Supplementary Fig. 9**

**Alignment of experimental and predicted structures of exostosin GT47 domains.** Models for EXT1, EXT2, and EXTL3 were downloaded from the AlphaFold Protein Structure Database. All structures were truncated to their GT47 domain prior to alignment to the experimental EXTL3 structure in PyMOL. The view on the right shows the conformation of the Cα4 helix.

```
              Nβ1                   Nα1                      Nβ4
              EEE-HHHHH---HHHHHHHHHHHHHHH---HHHHHH-----------EEEEE---------
HsEXTL3       VYVYDSDQFV--FGSYLDPLVKQAFQ---ATARANVYVTENADIACLYVILVGEMQEPVV (250)
CgEXT1        VYVYPQQK---------GEKIAESYQNILAAIEGSRFYTSDPSQACLFVLSLDTLDRDQL (166)
HsEXT1        VYVYPQQK---------GEKIAESYQNILAAIEGSRFYTSDPSQACLFVLSLDTLDRDQL (166)
HsEXT2        VYIYALKKYVDDFGVSVSNTISREYNELLMAISDSDYYTDDINRACLFVPSIDVLNQNTL (165)
              **:*  .:          .  :   ::    :   . : *.: . ***:*  :. ::   :

                   Nα4                Nβ5            Nα5        Nβ6     Nα6
              -H--HHHHHHHHH-----------EEEE----------HHHHHH-----EEEE--EE-HH
HsEXTL3       LR--PAELEKQLYSLPHWRTDGHNHVIINL-SRKSDTQNLLYNVSTGRAMVAQSTFYTVQ (307)
CgEXT1        SPQYVHNLRSKVQSLHLW-NNGRNHLIFNLYSGTWPDYTEDVGFDIGQQAMLAKASISTEN (225)
HsEXT1        SPQYVHNLRSKVQSLHLW-NNGRNHLIFNLYSGTWPDYTEDVGFDIGQQAMLAKASISTEN (225)
HsEXT2        R---IKETAQAMAQLSRW-DRGTNHLLFNMLPGGPPDYNTALDVPRDRALLAGGGFSTWT (221)
                    :  . : .*  *   * **:::*: .       .   ..  ..*::* . : *

                   Nβ7                            Cβ1
              H-----EE----------------HHHHH---------EEEEEE----------------
HsEXTL3       YRPGFDLVVSPLV---HAMS--EPNFMEIPPQVPVKRKYLFTFQGEK-IESLRSSLQEAR (361)
CgEXT1        FRPNFDVSI-PLFSKDHPRTGGERGFLKF-NTIPPLRKYMLVFKGKRYLTGIGSDTRNA- (282)
HsEXT1        FRPNFDVSI-PLFSKDHPRTGGERGFLKF-NTIPPLRKYMLVFKGKRYLTGIGSDTRNA- (282)
HsEXT2        YRQGYDVSI-PVY---SPLS----AEVDLPEKGPGPRQYFLLSS----QVGLHPEYRED- (268)
              :* .:*: : *:     . :      :.:    *  *:*::  .      .: .. .:

                                   Cα1         Cβ2
              -------------HHHHHHHHHHHHHHHH----EEE------------------------
HsEXTL3       SFEEEMEGDPPADYDDRIIATLKAVQDSKLDQVLVEFTCKNQPKPSLPTEWALC------ (415)
CgEXT1        ---------------------L--YHVHNGEDVVLLLTTCKHGKDWQKHKD-SRCDRDNTE (318)
HsEXT1        ---------------------L--YHVHNGEDVVLLLTTCKHGKDWQKHKD-SRCDRDNTE (318)
HsEXT2        ---------------------LEALQVKHGESVLVLDKCTNLSEGVLSVR-KRCHK---- (302)
                                   *   :  : :.*::  .*.:   .         *

                   Cα3        Cβ4                Cα4▼      Cβ5
              -HHHHHHHHHH-EEEEEEE------EEEHHHHHHHHHHHHHHH-EEEEE-----------
HsEXTL3       GEREDRLELLKLSTFALIITPGDPRLVISSGCATRLFEALEVGAVPVVLGEQVQLPYQDM (475)
CgEXT1        YEKYDYREMLHNATFCLV--PRGRRL-----GSFRFLEALQAACVPVMLSNGWELPFSEV (371)
HsEXT1        YEKYDYREMLHNATFCLV--PRGRRL-----GSFRFLEALQAACVPVMLSNGWELPFSEV (371)
HsEXT2        HQVFDYPQVLQEATFCVV--LRGARL-----GQAVLSDVLQAGCVPVVIADSYILPFSEV (355)
               :  *  ::*: :**.::    . **         : :.*:...***::.:   **:.::

                Cα5  Cβ6      Cα6             Cα7                Cα8
              -HHHHH-EEEHHHHHHHHHHHHHHHHHHHHHHHHHHHHHHHHHHHHHH-HHHHHHHHHHHHHH
HsEXTL3       LQWNEAALVVPKPRVTEVHFLLRSLSDSDLLAMRRQGRFLWETYFSTADSIFNTVLAMIR (535)
CgEXT1        INWNQAAVIGDERLLLQIPSTIRSIHQDKILALRQQTQFLWEAYFSSVEKIVLTTLEIIQ (431)
HsEXT1        INWNQAAVIGDERLLLQIPSTIRSIHQDKILALRQQTQFLWEAYFSSVEKIVLTTLEIIQ (431)
HsEXT2        LDWKRASVVVPEEKMSDVYSILQSIPQRQIEEMQRQARWFWEAYFQSIKAIALATLQIIN (415)
              ::*: *:::  :  : ::   :.*: : .:  :..* .::**:**.: . *   :.* :*.

              HHH
HsEXTL3       TRI (538)
CgEXT1        DRI (434)
HsEXT1        DRI (434)
HsEXT2        DRI (418)
               **
```

**Supplementary Fig. 10**

**Alignment of human EXT1, EXT2, EXTL1, and EXTL3 with Chinese hamster EXT1 (*Cg*EXT1).** Sequences were aligned using MUSCLE; the alignment was truncated to the GT47 domain. EXTL3 secondary structure is labelled above the sequences (H = α-helix, E = β-sheet). Residues (identified by Wei *et al.*[1]) whose mutation results in loss of *Cg*EXT1 glucuronosyltransferase activity are highlighted in black. The position of Arg340 in human EXT1 (most commonly mutated residue in hereditary multiple exostoses) is highlighted in grey. The conserved Glu/Asp on the Cα4 helix that we propose interacts with UDP-GlcA in EXT1 is marked with a black arrowhead.

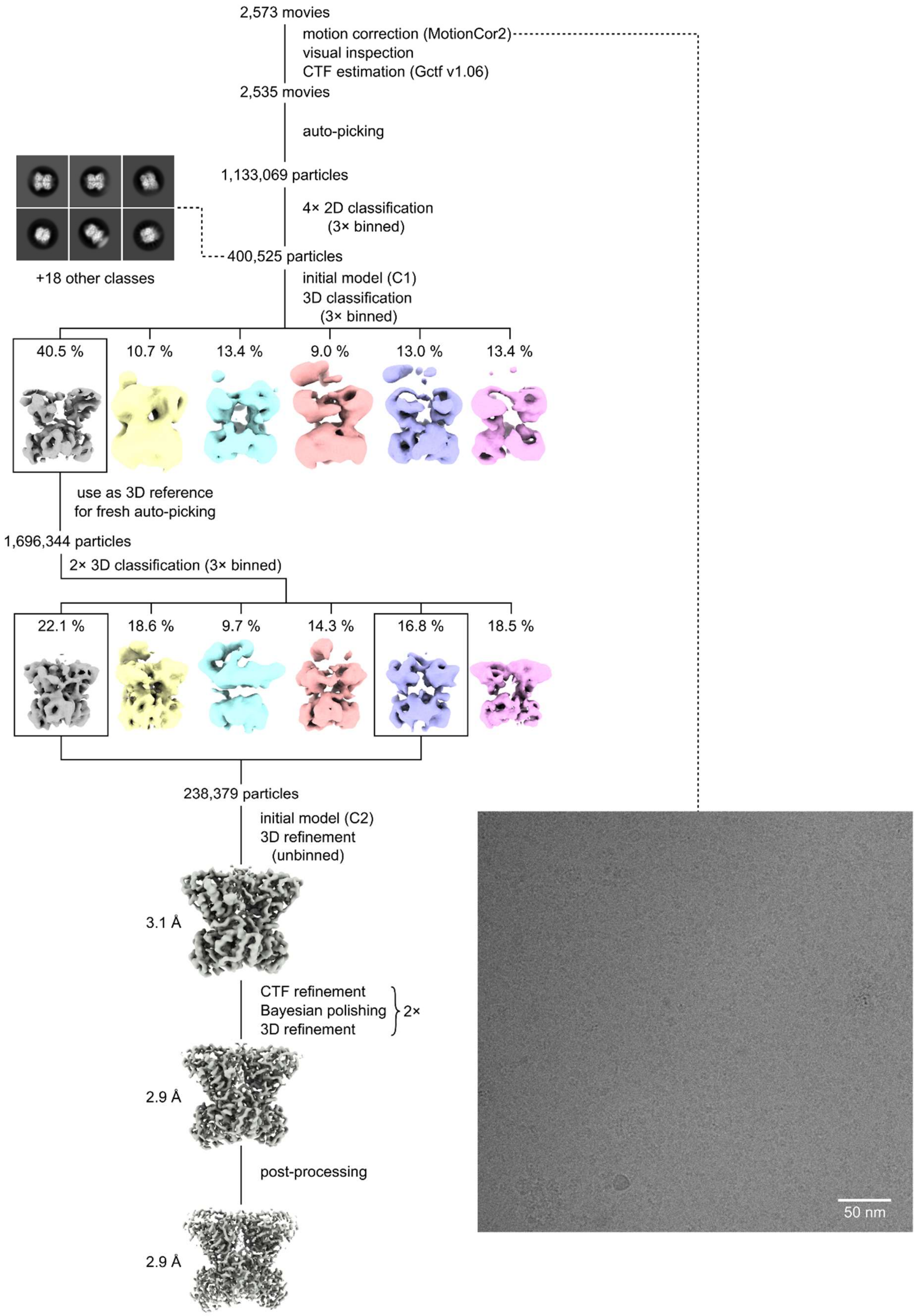


**Supplementary Fig. 11**

**Data processing pipeline for UDP-bound EXTL3 structure.** A representative motion-corrected micrograph is displayed on the right-hand side.

**a**

**b**

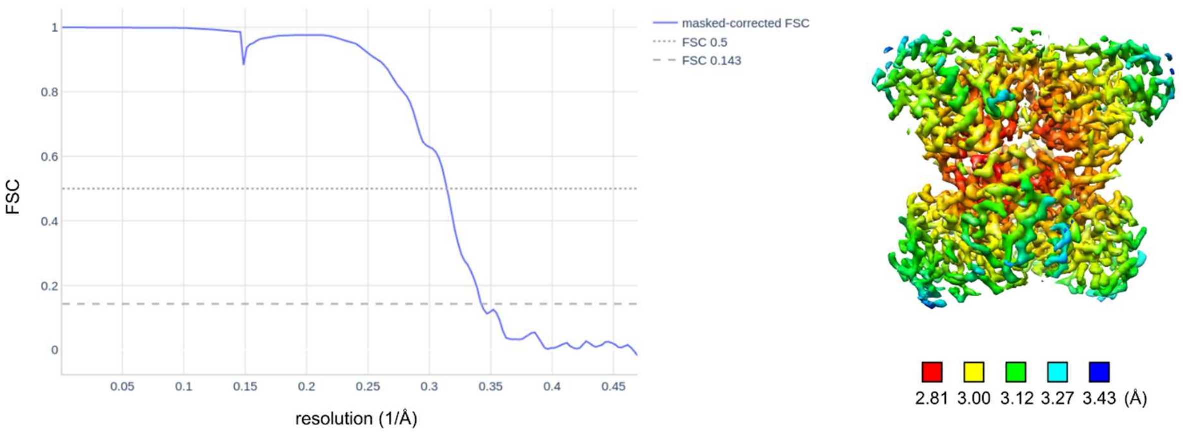


**Supplementary Fig. 12**

**Resolution estimates of the UDP-bound map. a** FSC curve. **b** Local resolution estimate, calculated using RELION, coloured from red (highest resolution) to blue (lowest resolution).

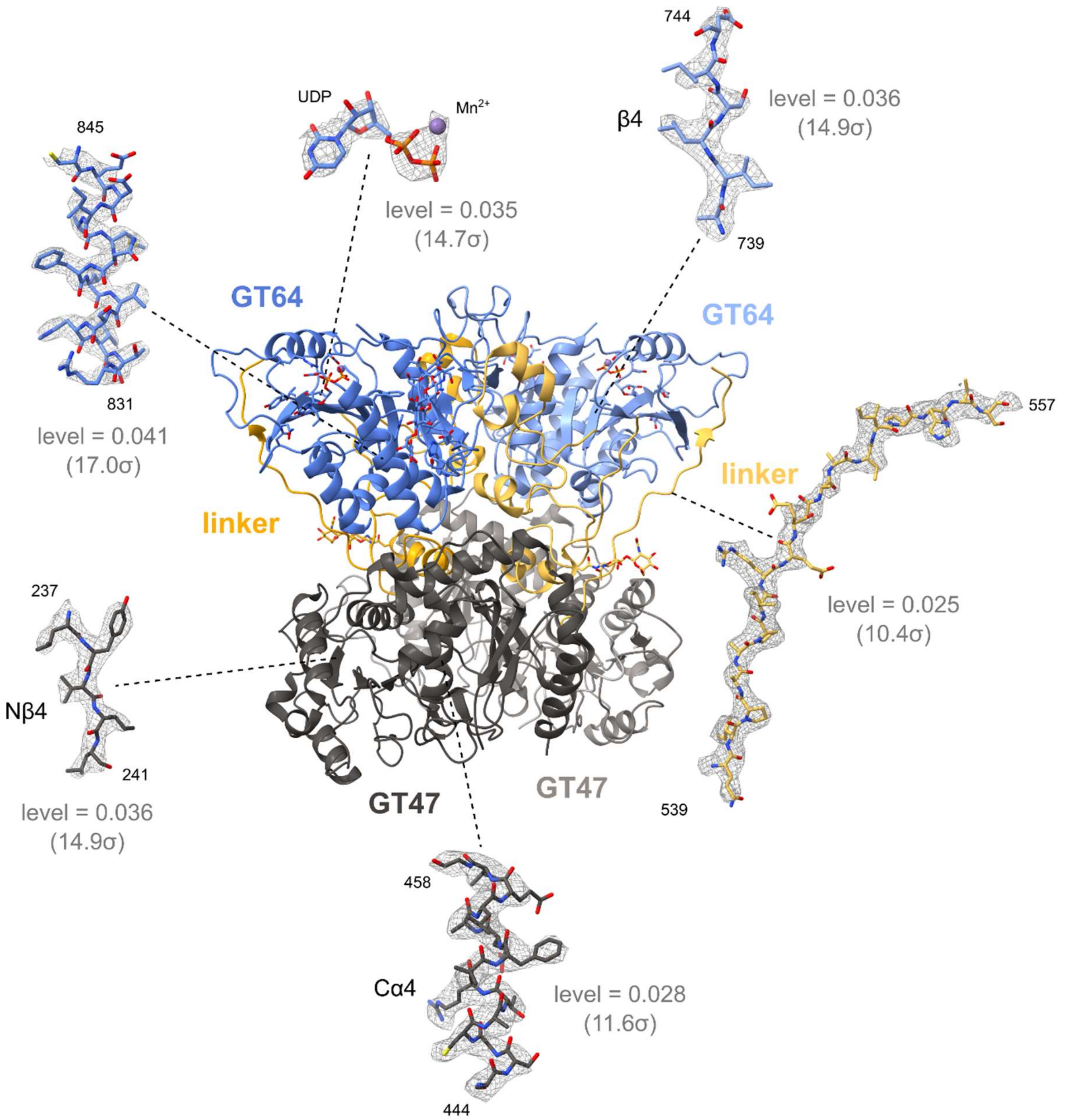


**Supplementary Fig. 13**

**Structure of UDP-bound EXTL3, showing map density in key regions.** GT47 domains: grey; linker regions: yellow; GT64 domains: blue. Each portion of the map is displayed at the indicated contour level.

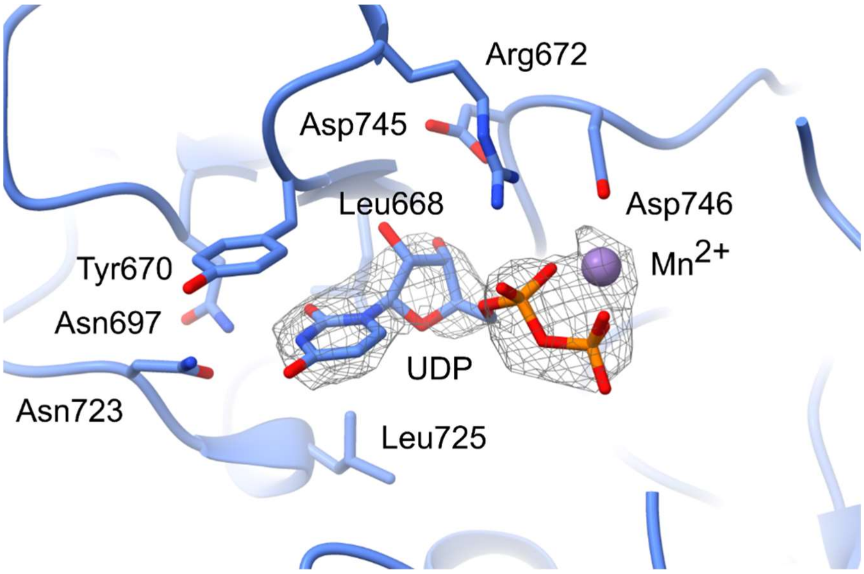


**Supplementary Fig. 14**

**EXTL3 UDP-binding site, showing density detected for UDP and $Mn^{2+}$.** Map density is shown at a contour level of 0.035 (14.7σ).

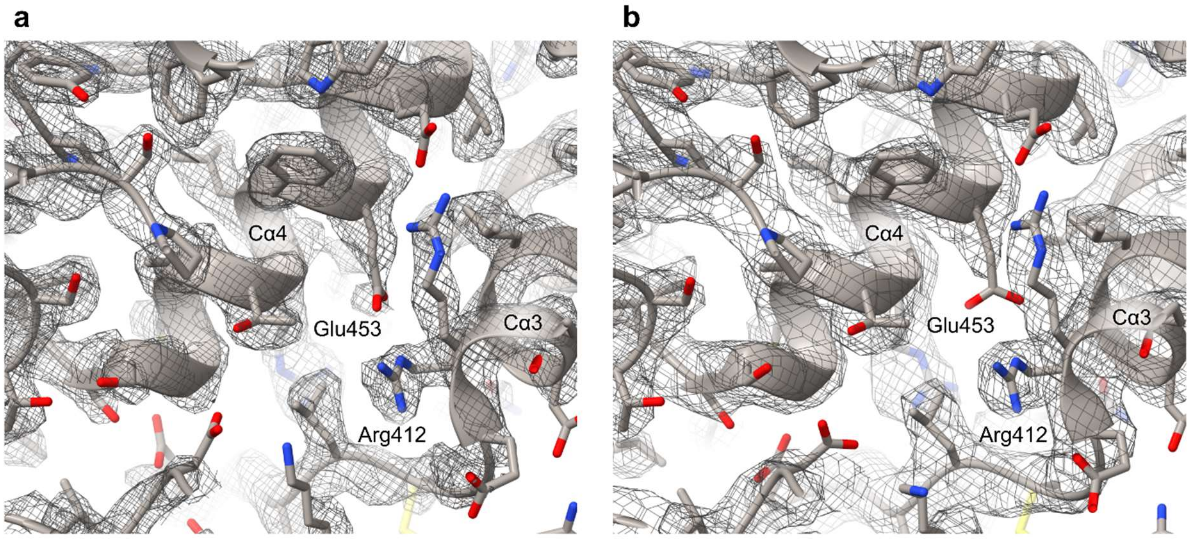


**Supplementary Fig. 15**

**No substrate binding or conformational change is apparent in the GT47 domain in the presence of UDP.** Comparison of cryo-EM map density in the predicted active site of the GT47 domain between **a** apo-structure (2.4 Å resolution; contour level = 0.050, 8.3σ) and **b** UDP-bound structure (2.9 Å resolution; contour level = 0.023, 9.6σ).

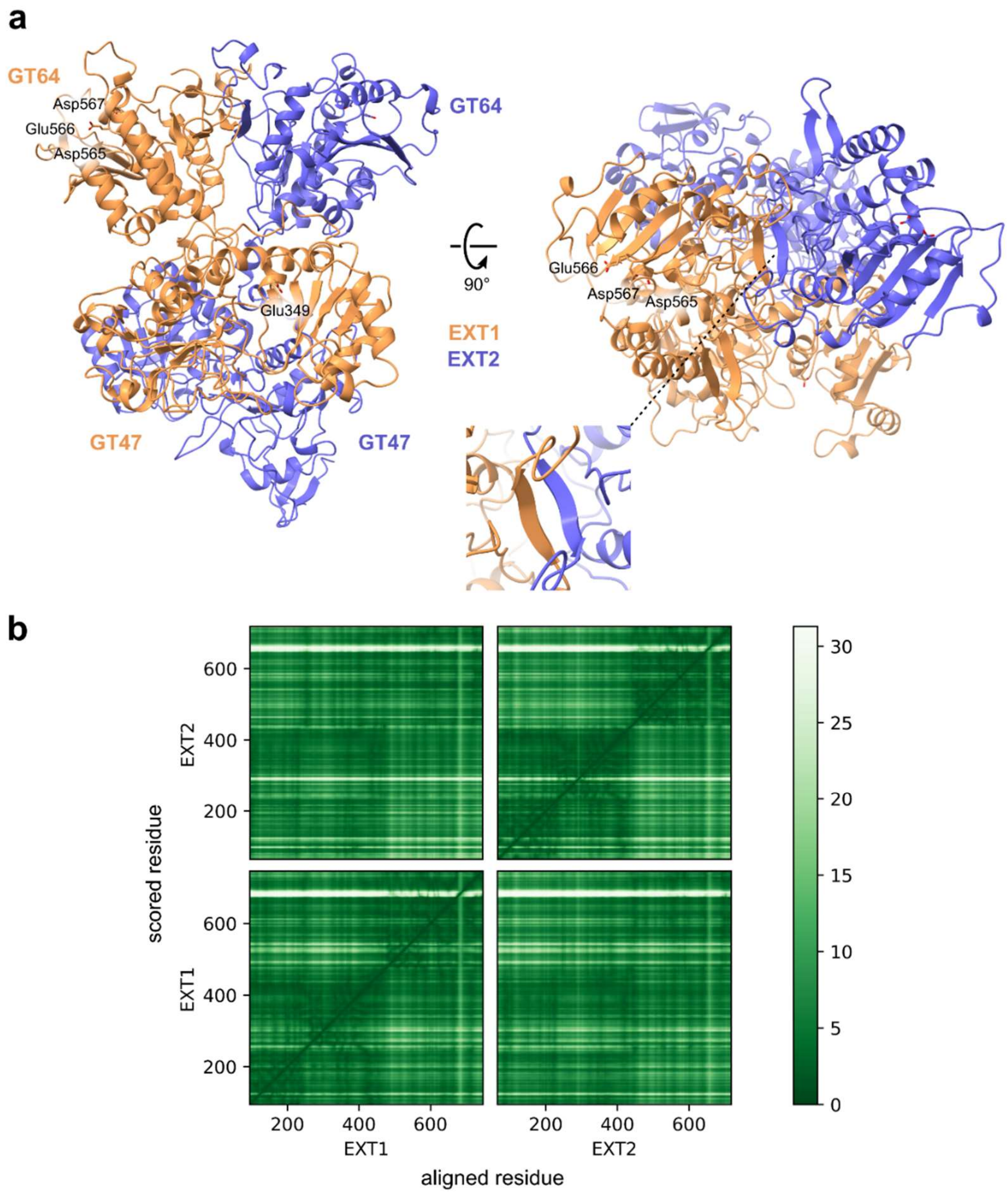


**Supplementary Fig. 16**

**Model of the EXT1/2 heterodimer created using AlphaFold-Multimer Colab.** Sequences corresponding to the globular domains of EXT1 (residues 95–746) and EXT2 (residues 65–718) were submitted to the AlphaFold-Multimer Colab server (https://colab.research.google.com/github/deepmind/alphafold/blob/main/notebooks/AlphaFold.ipynb). **a** The best model predicted by AlphaFold (EXT1 in orange; EXT2 in purple). The locations of residues putatively involved in binding UDP-GlcA (Glu349) or UDP-GlcNAc ($DED_{565–567}$) in EXT1 are marked. **b** Predicted aligned error (PAE) plot. Each point represents the PAE score (higher PAE = lower confidence in the relative position) between the predicted and true structures at residue *y* when the structures are aligned on residue *x*.

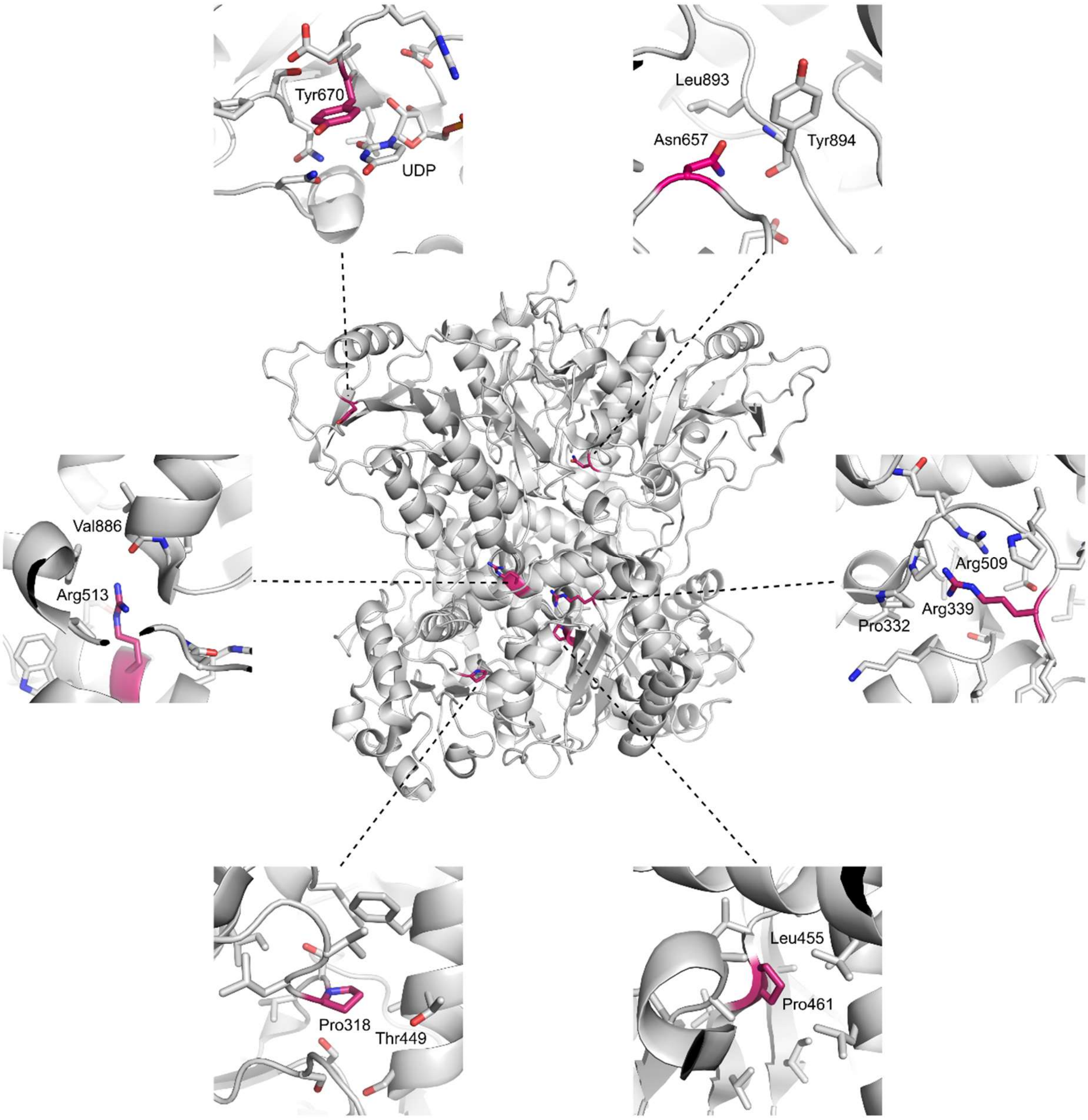


**Supplementary Fig. 17**

**Locations of characterised human missense mutations within the EXTL3 structure.** Residues for which deleterious mutations have been documented[2–4] (namely P318L, R339W, P461L, R513C, N657S, and Y670D) are shown in pink.

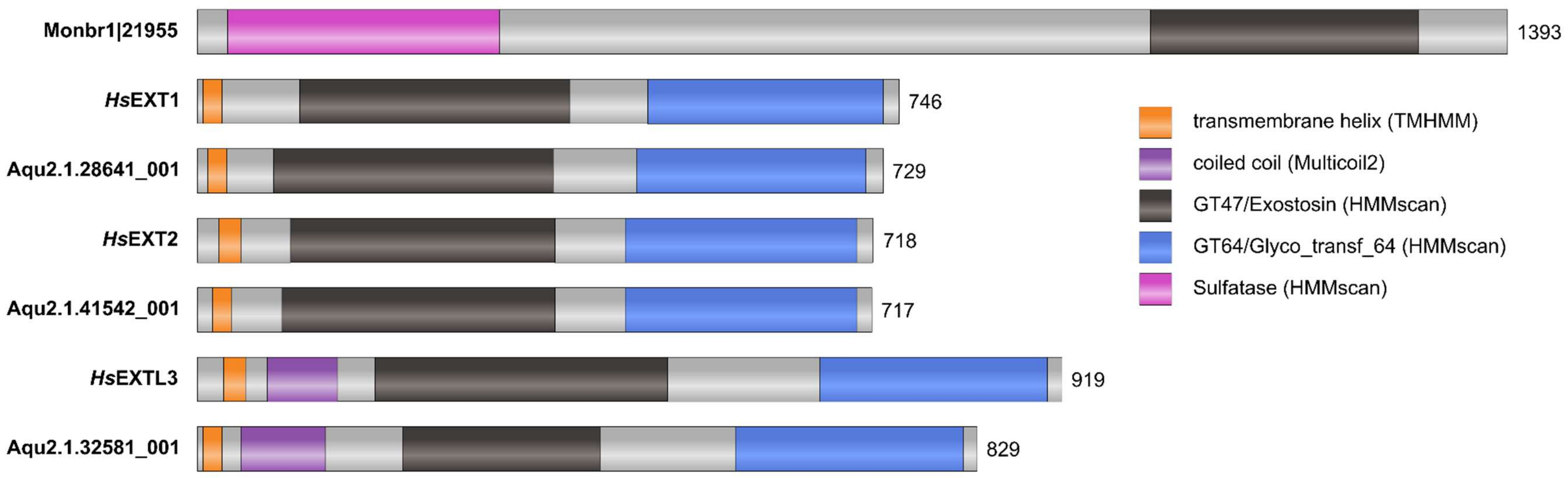


**Supplementary Fig. 18**

**Predicted domain structures of GT47-G (exostosin and exostosin-related) sequences from *Homo sapiens*, *Amphimedon queenslandica*, and *Monosiga brevicollis*.** Sequences from the GT47-G clade in Fig. 7 were submitted to the TMHMM[5] (https://services.healthtech.dtu.dk/service.php?TMHMM-2.0), Multicoil2[6] (http://cb.csail.mit.edu/cb/multicoil2/cgi-bin/multicoil2.cgi), and hmmscan[7,8] (https://www.ebi.ac.uk/Tools/hmmer/search/hmmscan) servers for prediction of transmembrane helices, coiled coils, and PFAM domains, respectively. The regions receiving hits for each are shown to scale.

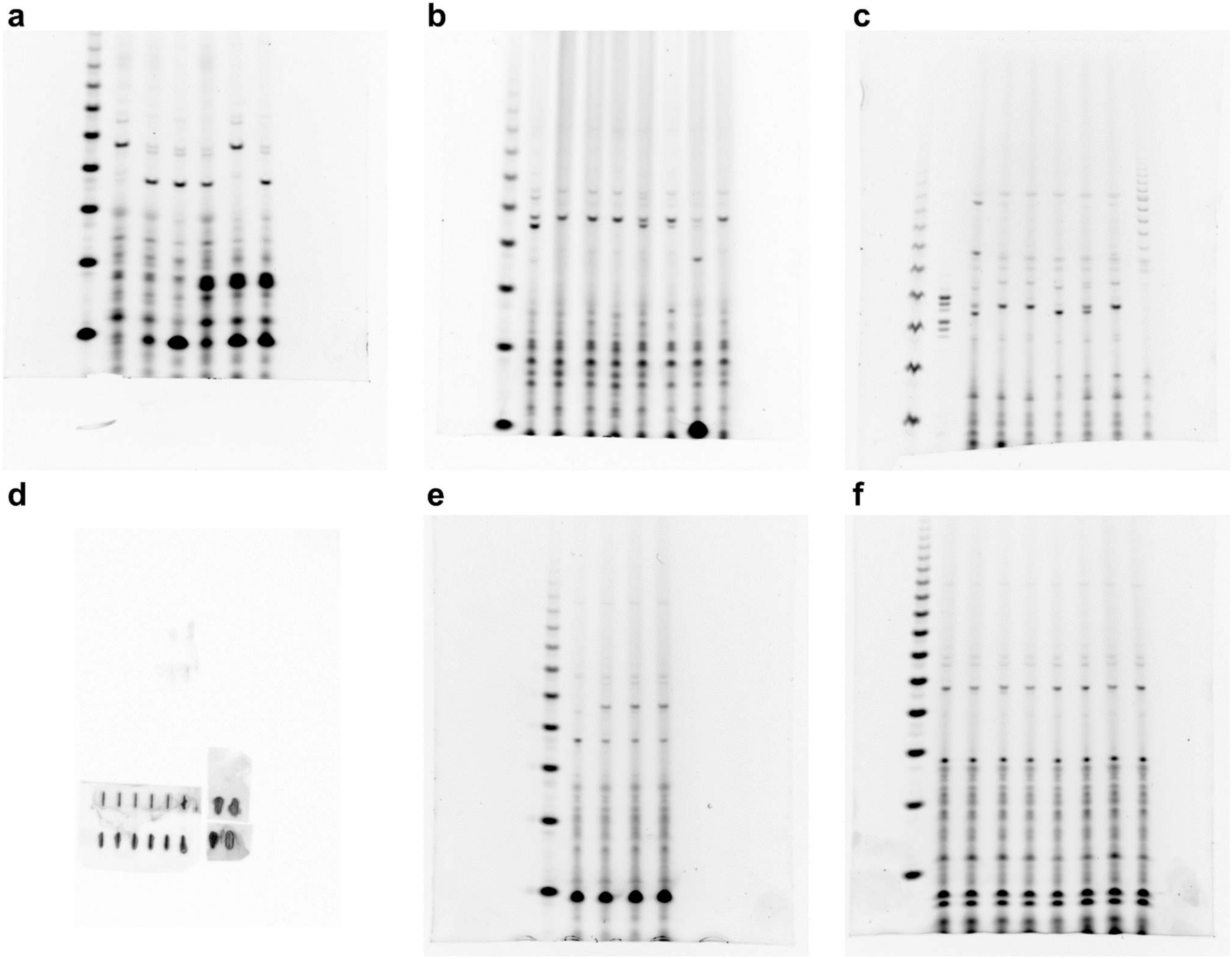

**Supplementary Fig. 19**

**Uncropped gels.** Uncropped gel images are provided for **a** Fig. 1b, **b** Fig. 1c, **c** Supplementary Fig. 1d, **d** Supplementary Fig. 3a, **e** Fig. 2b, and **f** Fig. 2c.

**Supplementary Table 1**

**Proteomic analysis of EXTL3ΔN preparation 1.** EXTL3ΔN was purified from the culture medium of EBNA 293 cells by nickel affinity chromatography and size exclusion. After concentration, an aliquot was treated with trypsin, and the resultant peptides were analysed by mass spectrometry. Protein abundances were quantified by label-free quantification. The top ten most abundant hits are shown along with the abundances for EXT1 and EXT2.

| **Rank** | **Protein name** | **Accession** | **Abundance (a.u.)** |
|---|---|---|---|
| 1 | EXTL3 | O34909 | $1.0 \times 10^{11}$ |
| 2 | Trypsin (*Sus scrofa*) | cRAP112 | $7.9 \times 10^{9}$ |
| 3 | Calsyntenin-1 | O94985 | $1.1 \times 10^{9}$ |
| 4 | Fibulin-1 | P23142 | $4.7 \times 10^{8}$ |
| 5 | LTBP4 isoform 2 | Q8N2S1-2 | $2.9 \times 10^{8}$ |
| 6 | Nidogen-1 | P14543 | $1.6 \times 10^{8}$ |
| 7 | GFP (*Aequorea victoria*) | cRAP032 | $1.5 \times 10^{8}$ |
| 8 | RAN | B5MDF5 | $1.5 \times 10^{8}$ |
| 9 | AHSG (*Bos taurus*) | cRAPR1 | $1.2 \times 10^{8}$ |
| 10 | PXDN | Q92626 | $1.1 \times 10^{8}$ |
| | … | | |
| 41 | EXT2 | Q93063 | $1.2 \times 10^{7}$ |
| | … | | |
| 78 | EXT1 | Q16394 | $1.6 \times 10^{6}$ |

**Supplementary Table 2**

**Proteomic analysis of EXTL3ΔN preparation 2.** EXTL3ΔN was purified from the culture medium of EBNA 293 cells by nickel affinity chromatography and size exclusion. After concentration, an aliquot was treated with trypsin, and the resultant peptides were analysed by mass spectrometry. Protein abundances were quantified by label-free quantification. The top ten most abundant hits are shown along with the abundances for EXT1 and EXT2.

| **Rank** | **Protein name** | **Accession** | **Abundance (a.u.)** |
|---|---|---|---|
| 1 | EXTL3 | O34909 | $1.0 \times 10^{12}$ |
| 2 | Trypsin (*Sus scrofa*) | cRAP112 | $3.2 \times 10^{10}$ |
| 3 | GLUD1 | P00367 | $2.1 \times 10^{10}$ |
| 4 | Actin, cytoplasmic 1 | P60709 | $2.6 \times 10^{9}$ |
| 5 | Clusterin | P10909 | $2.5 \times 10^{9}$ |
| 6 | HSPA1B | A0A0G2JIW1 | $2.5 \times 10^{9}$ |
| 7 | HSP90AA1 | P07900 | $2.0 \times 10^{9}$ |
| 8 | HTRA1 | Q92743 | $1.6 \times 10^{9}$ |
| 9 | LDHB | P07195 | $1.6 \times 10^{9}$ |
| 10 | LDHA | P00338 | $1.5 \times 10^{9}$ |
| | … | | |
| 89 | EXT2 | Q93063 | $1.2 \times 10^{8}$ |
| | … | | |
| 147 | EXT1 | Q16394 | $3.9 \times 10^{7}$ |

**Supplementary Table 3**

**Proteomic analysis of EXTL3ΔN preparations from EXTL3ΔN, EXTL3ΔN CRISPR$^{control}$, and EXTL3ΔN CRISPR$^{EXT1}$ cells.** EXTL3ΔN was purified from the culture media of EXTL3ΔN, EXTL3ΔN CRISPR$^{control}$ cells, and EXTL3ΔN CRISPR$^{EXT1}$ cells by nickel affinity chromatography and size exclusion. After concentration, an aliquot was treated with trypsin, and the resultant peptides were analysed by mass spectrometry. Protein abundances were quantified by label-free quantification. The top ten most abundant hits are shown along with the abundances for EXT1 and EXT2.

EXTL3ΔN

| Rank | Protein name | Accession | Abundance (a.u.) |
|---|---|---|---|
| 1 | EXTL3 | O34909 | $1.0 \times 10^{12}$ |
| 2 | Trypsin (*Sus scrofa*) | cRAP112 | $2.7 \times 10^{11}$ |
| 3 | NDNF | Q8TB73 | $6.7 \times 10^{9}$ |
| 4 | Calsyntenin-1 | O94985 | $5.4 \times 10^{9}$ |
| 5 | Trypsin-1 | E7EQ64 | $3.8 \times 10^{9}$ |
| 6 | Clusterin | P10909 | $3.1 \times 10^{9}$ |
| 7 | KRT1 | cRAP054 | $2.0 \times 10^{9}$ |
| 8 | Fibulin-1 | B1AHL2 | $8.2 \times 10^{8}$ |
| 9 | KRT16 | P08779 | $8.1 \times 10^{8}$ |
| 10 | PXDN | Q92626 | $8.1 \times 10^{8}$ |
| | … | | |
| 53 | EXT2 | Q93063 | $9.2 \times 10^{7}$ |
| | … | | |
| 87 | EXT1 | Q16394 | $2.1 \times 10^{7}$ |

EXTL3ΔN CRISPR$^{control}$

| Rank | Protein name | Accession | Abundance (a.u.) |
|---|---|---|---|
| 1 | EXTL3 | O34909 | $1.0 \times 10^{12}$ |
| 2 | Trypsin (*Sus scrofa*) | cRAP112 | $2.1 \times 10^{11}$ |
| 3 | Clusterin | P10909 | $1.5 \times 10^{9}$ |
| 4 | Trypsin-1 | E7EQ64 | $9.0 \times 10^{8}$ |
| 5 | AHSG (*Bos taurus*) | cRAPR1 | $7.0 \times 10^{8}$ |
| 6 | ADAMTS1 | Q9UHI8 | $6.5 \times 10^{8}$ |
| 7 | HIST1H2BK | O60814 | $6.0 \times 10^{8}$ |
| 8 | PXDN | Q92626 | $4.8 \times 10^{8}$ |
| 9 | HSPG2 | P98160 | $4.3 \times 10^{8}$ |
| 10 | ACTA1 | A6NL76 | $3.4 \times 10^{8}$ |
| | … | | |
| 15 | EXT2 | Q93063 | $2.1 \times 10^{8}$ |
| | … | | |
| 27 | EXT1 | Q16394 | $1.1 \times 10^{8}$ |

EXTL3ΔN CRISPR$^{EXT1}$

| Rank | Protein name | Accession | Abundance (a.u.) |
|---|---|---|---|
| 1 | EXTL3 | O34909 | $9.8 \times 10^{11}$ |
| 2 | Trypsin (*Sus scrofa*) | cRAP112 | $2.4 \times 10^{11}$ |
| 3 | KRT1 | cRAP054 | $1.1 \times 10^{9}$ |
| 4 | Clusterin | P10909 | $8.0 \times 10^{8}$ |
| 5 | ADAMTS1 | Q9UHI8 | $5.3 \times 10^{8}$ |
| 6 | AHSG (*Bos taurus*) | cRAPR1 | $5.2 \times 10^{8}$ |
| 7 | HIST1H2BK | O60814 | $4.2 \times 10^{8}$ |
| 8 | KRT9 | cRAP054 | $4.2 \times 10^{8}$ |
| 9 | PXDN | Q92626 | $3.6 \times 10^{8}$ |
| 10 | NELL2 | F8VVB6 | $3.2 \times 10^{8}$ |
| | … | | |
| 68 | EXT2 | Q93063 | $9.3 \times 10^{6}$ |
| | … | | |
| – | EXT1 | Q16394 | n.d. |

**Supplementary Table 4**

**Energetic contributions of EXTL3 domains to homodimerisation.** The structure was truncated to the following regions before submitting to the PISA server (https://www.ebi.ac.uk/msd-srv/prot_int/cgi-bin/piserver) to assess the individual energetic contribution of each part to homodimerisation.

| **Truncation** | **Residues** | **Free energy change upon homodimerization (kcal mol$^{-1}$)** |
|---|---|---|
| GT47 domain | 176–538 | −7.3 |
| linker region | 539–662 | −1.8 |
| GT64 domain | 663–919 | −18.8 |
| GT47 domain + linker | 176–662 | −23.7 |
| GT47 + GT64 | 176–538, 663–919 | −26.1 |
| linker + GT64 | 539–919 | −37.4 |
| full-length structure | 176–919 | −58.6 |

**Supplementary Table 5**

**Structures exhibiting similarity to the EXTL3 GT47 domain.** The fold of the GT47 domain was investigated by finding similar structures using the DALI server[9]. The whole GT47 domain, the N-terminal subdomain, and the C-terminal subdomain were each searched against the PDB25 database.

| **rank** | **PDB ID** | **protein name** | **species** | **GT family** | **rmsd** |
|---|---|---|---|---|---|
| **whole GT47 domain (residues 196–538)** | | | | | |
| 1 | 5F84 | POGLUT1 | *Drosophila melanogaster* | GT90 | 3.9 |
| 2 | 1IXY | DNA β-glucosyltransferase | T4 phage | GT63 | 3.8 |
| 3 | 4W6Q | GtfC | *Streptococcus agalactiae* | GT113 | 4.0 |
| 4 | 6EJI | PglH | *Campylobacter jejuni* | GT4 | 3.8 |
| 5 | 1XV5 | DNA α-glucosyltransferase | T4 phage | GT72 | 4.2 |
| **N-terminal subdomain (residues 196–324)** | | | | | |
| 1 | 4W6Q | GtfC | *Streptococcus agalactiae* | GT113 | 3.2 |
| 2 | 2W21 | DMDH | *Rhodotorula graminis* | non-GT | 3.0 |
| 3 | 6UUT | FabG | *Acinetobacter baumannii* | non-GT | 3.5 |
| 4 | 5MH5 | D2-HDH | *Haloferax mediterranei* | non-GT | 2.8 |
| 5 | 1YGY | PGDH | *Mycobacterium tuberculosis* | non-GT | 3.0 |
| **C-terminal subdomain (residues 342–522)** | | | | | |
| 1 | 5F84 | POGLUT1 | *Drosophila melanogaster* | GT90 | 2.9 |
| 2 | 4W6Q | GtfC | *Streptococcus agalactiae* | GT113 | 2.8 |
| 3 | 6EJI | PglH | *Campylobacter jejuni* | GT4 | 3.1 |
| 4 | 6TPK | glycogen synthase | *Pyrococcus abyssi* GE5 | GT5 | 2.8 |
| 5 | 2NZW | FucT | *Helicobacter pylori* | GT10 | 3.5 |

**Supplementary Table 6**

**Possible effects of disease-associated missense mutations in EXTL3 based on the EXTL3 structure.**

| Mutation | Possible effect of mutation |
|---|---|
| P318L | Steric clash between Leu318 side chain and Cα4 helix |
| R339W | Ablation of arginine pair interaction between Arg339 and Arg509 |
| P461L | Steric clash between Leu461 side chain and Cα4 helix |
| R513C | Ablation of hydrogen bond between Arg513 side chain and main-chain carbonyl of Val886; possible introduction of an inappropriate disulfide |
| N657S | Ablation of hydrogen bond(s) between Asn657 side chain and main chain of Tyr894 |
| Y670D | Reduction in binding affinity for uridine moiety of donor substrate |

**Supplementary Table 7**

**Data collection and refinement parameters for both apo- and UDP-bound maps.**

| | **apo-EXTL3 PDB: 7AU2 EMDB: 11923** | **UDP-bound EXTL3 PDB: 7AUA EMDB: 11926** |
|---|---|---|
| **Data collection and processing** | | |
| Detector | Falcon III | Gatan K2 |
| Nominal magnification | 120k | 130k |
| Energy filter slit size (eV) | none | 20 |
| Voltage (kV) | 300 | 300 |
| Total electron dose ($e^{-}$/Å$^2$) | 71.4 | 48.5 |
| Target defocus range (μm) | −3.0–−0.9 | −2.7–−1.2 |
| Calibrated pixel size (Å) | 0.667 | 1.065 |
| Movies collected | 1,440 | 2,573 |
| Symmetry imposed | *C*2 | *C*2 |
| Extraction box size (pixels) | 420 | 360 |
| Initial particle images (no.) | 656,292 | 1,696,344 |
| Final particle images (no.) | 171,285 | 238,379 |
| **Refinement** | | |
| Map resolution at FSC = 0.143 (Å) | 2.4 | 2.9 |
| Model composition | | |
| Non-hydrogen atoms | 11,236 | 11,300 |
| Protein residues | 1,372 | 1,372 |
| Non-protein atoms | 156 | 208 |
| *B* factors (Å$^2$) | | |
| Protein | 39.55 | 56.32 |
| Glycans/ligand | 46.21 | 68.81 |
| R.m.s. deviations | | |
| Bond lengths (Å) | 0.006 | 0.008 |
| Bond angles (°) | 1.043 | 0.982 |
| **Validation** | | |
| MolProbity score | 1.06 | 1.19 |
| Clashscore | 2.29 | 1.84 |
| Poor rotamers (%) | 0.83 | 0 |
| Ramachandran plot | | |
| Favored (%) | 97.79 | 96.31 |
| Allowed (%) | 2.21 | 3.69 |
| Disallowed (%) | 0 | 0 |

**Supplementary References**


1. Wei, G. *et al.* Location of the glucuronosyltransferase domain in the heparan sulfate copolymerase EXT1 by analysis of Chinese hamster ovary cell mutants. *J. Biol. Chem.* **275**, 27733–27740 (2000).

2. Guo, L. *et al.* Identification of biallelic EXTL3 mutations in a novel type of spondylo-epi-metaphyseal dysplasia. *J. Hum. Genet.* **62**, 797–801 (2017).

3. Oud, M. M. *et al.* Mutations in EXTL3 Cause Neuro-immuno-skeletal Dysplasia Syndrome. *Am. J. Hum. Genet.* **100**, 281–296 (2017).

4. Volpi, S. *et al.* EXTL3 mutations cause skeletal dysplasia, immune deficiency, and developmental delay. *J. Exp. Med.* **214**, 623–637 (2017).

5. Krogh, A., Rn Larsson, B. È., Von Heijne, G. & Sonnhammer, E. L. L. Predicting Transmembrane Protein Topology with a Hidden Markov Model: Application to Complete Genomes. *J. Mol. Biol.* **305**, 567–580 (2001).

6. Trigg, J., Gutwin, K., Keating, A. E. & Berger, B. Multicoil2: Predicting Coiled Coils and Their Oligomerization States from Sequence in the Twilight Zone. *PLoS One* **6**, e23519 (2011).

7. Eddy, S. R. Accelerated Profile HMM Searches. *PLoS Comput. Biol.* **7**, e1002195 (2011).

8. Potter, S. C. *et al.* HMMER web server: 2018 update. *Nucleic Acids Res.* **46**, W200–W204 (2018).

9. Holm, L. DALI and the persistence of protein shape. *Protein Sci.* **29**, 128–140 (2020).
