# Supplementary material for: The structure of EXTL3 helps to explain the different roles of bi-domain exostosins in heparan sulfate synthesis
Source: Nat Commun. 2022 Jun 8;13:3314. doi: 10.1038/s41467-022-31048-2 (PMC9178029; doi:10.1038/s41467-022-31048-2)
Supplement: Supplementary file 3 — Reporting Summary [file 41467_2022_31048_MOESM3_ESM.pdf]

Corresponding author(s): Katrin Mani
Paul Dupree

Last updated by author(s): May 17, 2022

Reporting Summary

Nature Portfolio wishes to improve the reproducibility of the work that we publish. This form provides structure for consistency and transparency in reporting. For further information on Nature Portfolio policies, see our Editorial Policies and the Editorial Policy Checklist.

Statistics

For all statistical analyses, confirm that the following items are present in the figure legend, table legend, main text, or Methods section.

n/a	Confirmed	
☐	☒	The exact sample size (*n*) for each experimental group/condition, given as a discrete number and unit of measurement
☐	☒	A statement on whether measurements were taken from distinct samples or whether the same sample was measured repeatedly
☐	☒	The statistical test(s) used AND whether they are one- or two-sided<br>*Only common tests should be described solely by name; describe more complex techniques in the Methods section.*
☒	☐	A description of all covariates tested
☒	☐	A description of any assumptions or corrections, such as tests of normality and adjustment for multiple comparisons
☐	☒	A full description of the statistical parameters including central tendency (e.g. means) or other basic estimates (e.g. regression coefficient) AND variation (e.g. standard deviation) or associated estimates of uncertainty (e.g. confidence intervals)
☐	☒	For null hypothesis testing, the test statistic (e.g. *F*, *t*, *r*) with confidence intervals, effect sizes, degrees of freedom and *P* value noted<br>*Give P values as exact values whenever suitable.*
☒	☐	For Bayesian analysis, information on the choice of priors and Markov chain Monte Carlo settings
☒	☐	For hierarchical and complex designs, identification of the appropriate level for tests and full reporting of outcomes
☒	☐	Estimates of effect sizes (e.g. Cohen's *d*, Pearson's *r*), indicating how they were calculated

Our web collection on statistics for biologists contains articles on many of the points above.

Software and code

Policy information about availability of computer code

Data collection	Bruker flexControl 3.4<br>GeneSnap 7.12
Data analysis	RELION 3.0.1<br>MotionCor2<br>Gctf v1.06<br>CCPEM 1.4.1<br>Buccaneer (version n/a)<br>Coot 0.8.9.2<br>Phenix 1.18.2-3874<br>ISOLDE 1.1<br>ChimeraX 1.1<br>PyMOL 2.4.0a0 Open-Source<br>Protein Discoverer version 2.3<br>Mascot search engine 2.6.0<br>Scaffold 4.10.0<br>Bruker flexAnalysis 3.4<br>ImageJ 1.51q<br>HMMER 3.1b2<br>RAxML 8.2.11<br>FigTree v1.4.4<br>MUSCLE v3.8.31

MAFFT v7.310
ProtTest3 3.4.2
Python 2.7.17
Inkscape 1.0.1
DALI server (http://ekhidna2.biocenter.helsinki.fi/dali/; version n/a)
AlphaFold Colab server (https://colab.research.google.com/github/deepmind/alphafold/blob/main/notebooks/AlphaFold.ipynb; simplified version of v2.1.0)
TMHMM server (https://services.healthtech.dtu.dk/service.php?TMHMM-2.0, version 2.0)
Multicoil2 server (http://cb.csail.mit.edu/cb/multicoil2/cgi-bin/multicoil2.cgi, version n/a)
hmmscan search at the HMMER server (https://www.ebi.ac.uk/Tools/hmmer/search/hmmscan, version 2.41.2)
Custom code: alitrunc.py Python script, available at https://doi.org/10.5281/zenodo.6562402

For manuscripts utilizing custom algorithms or software that are central to the research but not yet described in published literature, software must be made available to editors and reviewers. We strongly encourage code deposition in a community repository (e.g. GitHub). See the Nature Portfolio guidelines for submitting code & software for further information.

Data

Policy information about availability of data

All manuscripts must include a data availability statement. This statement should provide the following information, where applicable:

- Accession codes, unique identifiers, or web links for publicly available datasets
- A description of any restrictions on data availability
- For clinical datasets or third party data, please ensure that the statement adheres to our policy

Cryo-EM maps for apo¬- and UDP-bound EXTL3 were deposited in the Electron Microscopy Data Bank under accession codes EMD-11923 [https://www.ebi.ac.uk/PDBe/entry/EMDB/11923] (apo structure) and EMD-11926 [https://www.ebi.ac.uk/PDBe/entry/EMDB/11926] (UDP-bound structure). Atomic co-ordinates for apo- and UDP-bound EXTL3 were submitted to the Protein Data Bank under accession codes 7AU2 [https://www.rcsb.org/structure/7AU2] (apo structure) and 7AUA [https://www.rcsb.org/structure/7AUA] (UDP-bound structure). The mass spectrometry proteomics data have been deposited to the ProteomeXchange Consortium via the PRIDE93 partner repository with the dataset identifiers PXD032145 [http://proteomecentral.proteomexchange.org/cgi/GetDataset?ID=PXD032145] (regular EXTL3ΔN purification) and PXD032144 [http://proteomecentral.proteomexchange.org/cgi/GetDataset?ID=PXD032144] (CRISPR experiments). Proteomic search databases are available at UniProt (human proteome reference [https://www.uniprot.org/proteomes/UP000005640]) and The Global Proteome Machine (cRAP common contaminants database [https://www.thegpm.org/crap/]). Numerical data for Fig. 2d,e, Supplementary Fig. 2e, and Supplementary Fig. 3b can be found in the Source Data file. Individual protein sequences were downloaded from the UniProtKB (HsEXT1: Q16394 [https://www.uniprot.org/uniprot/Q16394]; HsEXT2: Q93063 [https://www.uniprot.org/uniprot/Q93063]; HsEXTL1: Q92935 [https://www.uniprot.org/uniprot/Q92935]; HsEXTL3: O43909 [https://www.uniprot.org/uniprot/O43909]) or TAIR (AtMUR3: AT2G20370 [https://www.arabidopsis.org/servlets/TairObject?id=31312&type=locus]; AtXLT2: AT5G62220 [https://www.arabidopsis.org/servlets/TairObject?id=133787&type=locus]; AtXUT1: AT1G63450 [https://www.arabidopsis.org/servlets/TairObject?id=30091&type=locus]; AtARAD1: AT2G35100 [https://www.arabidopsis.org/servlets/TairObject?id=35143&type=locus]; AtIRX10: AT1G27440 [https://www.arabidopsis.org/servlets/TairObject?id=28129&type=locus]; AtIRX10L: AT5G61840 [https://www.arabidopsis.org/servlets/TairObject?id=132614&type=locus]; AtIRX7: AT2G28110 [https://www.arabidopsis.org/servlets/TairObject?id=32515&type=locus]; AtIRX7L: AT5G22940 [https://www.arabidopsis.org/servlets/TairObject?id=134444&type=locus]; AtExAD: AT3G57630 [https://www.arabidopsis.org/servlets/TairObject?id=36016&type=locus]; AtXGD1: AT5G33290 [https://www.arabidopsis.org/servlets/TairObject?id=130832&type=locus]) databases. AlphaFold pre-computed structural predictions are available from the AlphaFold Protein Structure Database (HsEXT1: Q16394 [https://alphafold.ebi.ac.uk/entry/Q16394]; HsEXT2: Q93063 [https://alphafold.ebi.ac.uk/entry/Q93063]; HsEXTL3: O43909 [https://alphafold.ebi.ac.uk/entry/O43909]). Proteome models are available at NCBI Genome (Homo sapiens: RefSeq GCF_000001405.40 [https://www.ncbi.nlm.nih.gov/data-hub/genome/GCF_000001405.40/]; Drosophila melanogaster: RefSeq GCF_000001215.4 [https://www.ncbi.nlm.nih.gov/data-hub/genome/GCF_000001215.4/]; Caenorhabditis elegans: RefSeq GCF_000002985.6 [https://www.ncbi.nlm.nih.gov/data-hub/genome/GCF_000002985.6/]), JGI Phytozome (Arabidopsis thaliana: 167 [https://phytozome-next.jgi.doe.gov/info/Athaliana_TAIR10]; Physcomitrium patens: 318 [https://phytozome-next.jgi.doe.gov/info/Ppatens_v3_3]), JGI MycoCosm (Monosiga brevicollis: Monosiga brevicollis MX1 [https://mycocosm.jgi.doe.gov/Monbr1/Monbr1.home.html]), EnsemblMetazoa (Amphimedon queenslandica: Aqu1 [https://metazoa.ensembl.org/Amphimedon_queenslandica/Info/Index]), and GigaDB (Ginkgo biloba: 100613 [http://gigadb.org/dataset/100613]). The GT47 Hidden Markov Model used in this work is available from the dbCAN2 server (dbCAN-HMMdb-V9 [https://bcb.unl.edu/dbCAN2/download/dbCAN-HMMdb-V9.txt]).

Field-specific reporting

Please select the one below that is the best fit for your research. If you are not sure, read the appropriate sections before making your selection.

☒ Life sciences ☐ Behavioural & social sciences ☐ Ecological, evolutionary & environmental sciences

For a reference copy of the document with all sections, see nature.com/documents/nr-reporting-summary-flat.pdf

Life sciences study design

All studies must disclose on these points even when the disclosure is negative.

Sample size	No sample size calculation was performed. Sample sizes were determined by feasibility, and are justified by the low variance within each group.
Data exclusions	There were no data exclusions.

Replication	Two successful independent replicates were performed for overnight GlcNAc-T assays, GlcA-T assays, immunofluorescence, and slot blotting (Figs. 1b, 1c, Supplementary Figs. 1, 2, 3.) For CRISPR GlcNAc-T/GlcA-T assays (Fig. 2), a second fully independent replicate was not attempted.
Randomization	Allocation is not relevant to our study. The samples/cells are not expected to exhibit any significant covariates.
Blinding	For immunofluorescence samples both replicates were also analysed by a blinded expert in fluorescence microscopy. None of the other experiments involved a subjective element, hence blinding was irrelevant.

Reporting for specific materials, systems and methods

We require information from authors about some types of materials, experimental systems and methods used in many studies. Here, indicate whether each material, system or method listed is relevant to your study. If you are not sure if a list item applies to your research, read the appropriate section before selecting a response.

Materials & experimental systems

n/a	Involved in the study
☐	☒ Antibodies
☐	☒ Eukaryotic cell lines
☒	☐ Palaeontology and archaeology
☒	☐ Animals and other organisms
☒	☐ Human research participants
☒	☐ Clinical data
☒	☐ Dual use research of concern

Methods

n/a	Involved in the study
☒	☐ ChIP-seq
☒	☐ Flow cytometry
☒	☐ MRI-based neuroimaging

Antibodies

Antibodies used	EXT-1 (A-7): sc-515144, Lot # K0316 (SANTA CRUZ BIOTECHNOLOGY); Sigma-Aldrich Anti beta-actin: A3854, lot # 089M4850V (Sigma-Aldrich); horseradish peroxidase-conjugated anti-mouse IgG (Cat. nr. 172-1011; Bio-Rad), lot # L006326 A; Alexa Fluor 594-tagged goat anti-mouse IgG (Cat. nr. A-11005; Molecular Probes / ThermoFisher), lot #84B3-1
Validation	EXT-1 (A-7): sc-515144 was validated for Western Blotting and immunofluorescence by the manufacturer (https://datasheets.scbt.com/sc-515144.pdf).<br>Anti beta-actin: A3854 was validated for Western Blotting by the manufacturer (https://www.sigmaaldrich.com/SE/en/product/sigma/a3854).<br>Anti-mouse IgG: 1721011 was validated for Western Blotting by the manufacturer (https://www.bio-rad-antibodies.com/polyclonal/mouse-igg-antibody-star207.html)<br>Alexa Fluor 594-tagged goat anti-mouse IgG was validated for immunofluorescence by the manufacturer (https://www.thermofisher.com/order/genome-database/dataSheetPdf?producttype=antibody&productsubtype=antibody_secondary&productId=A-11005&version=225)

Eukaryotic cell lines

Policy information about cell lines

Cell line source(s)	EBNA 293 cells were originated from ATCC
Authentication	The cell line was not authenticated.
Mycoplasma contamination	Cell lines were routinely treated with mycoplasma removing agent from MP biomedicals (Cat. nr. 3050044, Lot#3S030) at the concentration of 0.5 µg/ml for one week after thawing. No mycoplasma contamination was expected.
Commonly misidentified lines (See ICLAC register)	No commonly misidentified lines were used.
